# Expression of the Wnt Receptor Frizzled-4 in the Human Enteric Nervous System of Infants

**DOI:** 10.1155/2016/9076823

**Published:** 2015-11-30

**Authors:** Katharina Nothelfer, Florian Obermayr, Nadine Belz, Ellen Reinartz, Petra M. Bareiss, Hans-Jörg Bühring, Rudi Beschorner, Lothar Just

**Affiliations:** ^1^Institute of Clinical Anatomy and Cell Analysis, University of Tübingen, Tübingen, Germany; ^2^Department of Pediatric Surgery and Pediatric Urology, University Children's Hospital Tübingen, Tübingen, Germany; ^3^Division of Hematology, Immunology, Oncology, Rheumatology and Pulmonology, Department of Internal Medicine II, University of Tübingen, Tübingen, Germany; ^4^Institute for Pathology and Neuropathology, Department of Neuropathology, University of Tübingen, Tübingen, Germany

## Abstract

The Wnt signalling pathway plays a crucial role in the development of the nervous system. This signalling cascade is initiated upon binding of the secreted Wnt ligand to a member of the family of frizzled receptors. In the present study, we analysed the presence of frizzled-4 in the enteric nervous system of human infants. Frizzled-4 could be identified by immunohistochemistry in a subpopulation of enteric neuronal and glial cells in the small and large intestine. Detection of frizzled-4 in the tunica muscularis by RT-PCR confirmed this receptor's expression on the mRNA level. Interestingly, we observed distinct cell populations that co-expressed frizzled-4 with the intermediate filament protein nestin and the neurotrophin receptor p75^NTR^, which have been reported to be expressed in neural progenitor cells. Flow cytometry analysis revealed that 60% of p75^NTR^ positive cells of the tunica muscularis were positive for frizzled-4. Additionally, in pathological samples of Hirschsprung's disease, the expression of this Wnt receptor correlated with the number of myenteric ganglion cells and decreased from normoganglionic to aganglionic areas of large intestine. The expression pattern of frizzled-4 indicates that this Wnt receptor could be involved in postnatal development and/or function of the enteric nervous system.

## 1. Introduction

Neurons and glial cells of the peripheral nervous system derive from neural crest stem cells (NCSCs) [1]. Following a distinct and well defined spatiotemporal pattern, NCSCs migrate out of the neural tube into embryonic tissue, including the developing gut. They enter the primitive foregut by embryonic day (E) 9.5 in mice and migrate in a rostral-to-caudal direction in order to colonize the entire gut by E14.5 [2, 3]. In humans migration of enteric neural crest cells takes place between gestational week 4 and gestational week 7 [4]. During their journey along the gut a proportion of cells continues to proliferate, while others begin to differentiate [5] into neurons and glial cells in order to generate a complex neural network that coordinates bowel motility and is involved in regulation of its secretory activity, blood flow, and modulation of the immune system [6, 7]. Impairment of ENS development leads to incomplete colonization of the gut by NCSCs, resulting in peristaltic dysregulation, intestinal obstruction, and enterocolitis as observed in Hirschsprung's disease (HSCR) [8–10]. Much effort has been put into research investigating the genetic regulation of neural crest development and its derivatives during the last decades. This resulted in the identification of several major pathways regulating NCSC induction, migration, differentiation, and interconnection of developing neurons and glial cells [3, 11]. Wnt signalling has been reported to play a central role in these developmental processes in many model organisms. In concert with members of the bone morphogenic protein (BMP) family and fibroblast growth factors, Wnts regulate neural crest induction and specification. In addition, migration of neural crest cells is dependent on the Wnt signalling pathway [12, 13].

The Wnt signalling cascade is initiated by binding of Wnt to members of the frizzled transmembrane receptor family and results in activation of pathways like the *β*-catenin-dependent pathway or the planar cell polarity- (PCP-) dependent pathway, both of which have been shown to be involved in early neural crest development. Recently, the PCP pathway has been demonstrated to be additionally involved in regulating neural interconnection during ENS development [14]. However, while the importance of Wnt-frizzled interaction is well established for early neural crest development, its role in regulating postnatal neural crest derivatives is largely unknown.

Interestingly, there is evidence that neurogenesis continues in postnatal mice* in vivo* upon stimulation with serotonin or following injury [15–17]. A subpopulation of cells of the postnatal ENS has been attributed with stem cell like properties, although this niche is not well characterized yet. Nestin, the low-affinity nerve growth factor receptor p75^NTR^, and CD49b (alpha2-integrin) have been suggested to represent markers for this population of enteric neural stem and progenitor cells [18].

Of the frizzled receptor family members, frizzled-4 has previously been shown to be of particular interest with respect to its role in the regulation of stem cell maintenance, neuroprotection, and cell migration in the CNS [19–21]. In the present study, we examined the expression of this Wnt receptor in the enteric nervous system of infants by immunohistochemistry, RT-PCR, and flow cytometry analysis. In order to characterize the frizzled-4 positive cell population we performed colabelling experiments with various neural markers that are expressed in neural and putative progenitor cells of the ENS.

## 2. Materials and Methods 

### 2.1. Human Gut Samples

Human gut samples from small and large intestine were obtained from infant male and female patients at the age of 4 weeks to 10 months, who underwent surgical enterostoma removal after treatment for necrotizing enterocolitis or anorectal malformations. In addition, tissue was collected from Hirschsprung's disease patients, who underwent a transanal endorectal pull-through procedure (Table 1). All samples were collected according to the guidelines and after approval of the Local Ethical Committee at the University of Tübingen and with the consent of the patient's parents.

### 2.2. Immunohistochemistry

Immunohistochemistry was performed on 14 *μ*m thick cryosections. Tissue slides were fixed in 4% (w/v) paraformaldehyde in phosphate-buffered saline (PBS, pH 7.4) for 20 min at room temperature and subsequently rinsed three times in PBS. Slides, which were stained with the ABC-detection system (Vector Labs, Peterborough, UK), were additionally pretreated in 3% (v/v) hydrogen peroxide PBS solution for 10 min to block endogenous peroxidases. After incubation with serum containing blocking buffer (PBS containing 0.1% (w/v) BSA, 4% (v/v) goat serum, and 0.3% (v/v) Triton X-100) for 30 min, sections were incubated with primary antibodies diluted in PBS, 0.1% (w/v) BSA, and 0.1% (v/v) Triton X-100 overnight at 4°C. Primary antibodies and concentrations are listed in Table 2. Following three washes with PBS, the sections were incubated with secondary antibodies for 30 min at room temperature in the same buffer. Detection was performed with either fluorochrome-conjugated secondary antibodies (1 : 400, goat anti-rabbit IgG-Cy3 (Dianova, Hamburg, Germany); goat anti-mouse IgG-Cy3 (Dianova, Hamburg, Germany); goat anti-mouse IgG-Alexa 488 (Life Technologies, Darmstadt, Germany)) or biotinylated secondary antibodies (1 : 400, swine anti-rabbit IgG-biotin and rabbit anti-mouse IgG-biotin (Dako, Hamburg, Germany)) when using the ABC-detection system. Incubation of the AB reagent was performed according to the manufacturer and visualization of the ABC system was carried out with 3-3′diaminobenzidine (DAB, Sigma, Taufkirchen, Germany) and hydrogen peroxide solution. For fluorescence detection, the sections and cells were washed three times in PBS and additionally stained with DAPI (4′-6-diamidino-2-phenylindole, 0.2 *μ*g/mL) PBS solution for 10 min. Stained sections were dried, embedded in Kaiser's gelatin (Merck, Darmstadt, Germany), and photographed on an inverted microscope (Axiovert, Zeiss, Jena, Germany).

### 2.3. RNA Isolation and RT-PCR

Human gut samples were mechanically dissected with the visual aid of a stereomicroscope. After disposure of residual fat and mesenteric tissue, the tunica muscularis was peeled off the submucosal and mucosal layers and thoroughly minced. Total RNA was isolated from human tunica muscularis using the RNeasy Mini Kit (Qiagen, Hilden, Germany) according to the manufacturer's instructions. Possible contaminating genomic DNA was removed by treatment with DNase I (Life Technologies, Darmstadt, Germany). Reverse transcriptase PCR (RT-PCR) reaction containing oligo-(dT)_23_ anchored mRNA primers (Sigma-Aldrich, Taufkirchen, Germany), Superscript II Reverse Transcriptase enzyme, and RNase Out was performed according to the supplier's protocol (Life Technologies, Darmstadt, Germany).

To amplify the frizzled-4 receptor and the housekeeping gene GAPDH, the following primer pairs (Sigma-Aldrich, Taufkirchen, Germany) were used.

 Frizzled-4 (product size: 605 bp):
   5′-CTCGGCTACAACGTGACCAAGAT-3′,
   5′-AATATGATGGGGCGCTCAGGGTA-3′;
 GAPDH (product size: 452 bp):
   5′-ACCACAGTCCATGCCATCAC-3′,
   5′-TCCACCACCCTGTTGCTGTA-3′.

The PCR reaction mixture was incubated at 95°C for 5 min, followed by 35 cycles of 95°C for 30 s, annealing temperatures (*T*_An_) for 40 s and 72°C for 40 s, and a final cycle with a prolonged elongation time of 10 min at 72°C. The primer-specific annealing temperatures were as follows: *T*_An_ (frizzled-4) = 58°C and *T*_An_ (GAPDH) = 60°C. Amplified PCR products were analyzed by electrophoresis on a 1% (w/v) agarose gel (Roth, Karlsruhe, Germany) in 1x TBE buffer (Tris base, boric acid, and EDTA) at 100 V. The products were visualized with ethidium bromide (2 *μ*g/mL) on U.V. light. The size of each PCR product was estimated by using a 100 bp DNA ladder standard (Life Technologies, Darmstadt, Germany). The RT step was omitted as a control for possible contamination of DNase-treated samples with residual genomic DNA (negative control).

### 2.4. Flow Cytometry

Cells used for flow cytometry analysis were obtained from human tissue by digestion of the tunica muscularis with 750 U/mL Collagenase XI (Sigma, Taufkirchen, Germany) and 0.5% w/v Dispase II (Roche, Mannheim, Germany) in HBSS with Ca^2+^/Mg^2+^. The digestion was carried out for a maximum of 1 h at 37°C, in order to avoid digestion of surface epitopes. Tissue residues were discarded and the supernatant was centrifuged for 7 min at 210 g, washed twice with HBSS, and filtered through a 40 *μ*m cell strainer. The resulting cell pellet was resuspended in 20 *μ*L blocking buffer (polyglobin (Talecris Biotherapeutics, Frankfurt am Main, Germany) diluted 1 : 10 in DPBS/0.5% BSA (0.5% (w/v) BSA in Dulbecco's PBS without Ca^2+^/Mg^2+^)) and incubated on ice for 10 min. Subsequently, cells were diluted with DPBS/0.5% (w/v) BSA to a concentration of 2 × 10^5^ cells per 50 *μ*L and antibodies were added for 15 min on ice. P75^NTR^ was detected with rabbit anti-p75^NTR^-APC (CD271, Miltenyi, Bergisch Gladbach, Germany, 1 : 50). Staining of frizzled-4 was achieved either by incubation with a mouse anti-frizzled-4 antibody (25 *μ*L/10^4^ cells, undiluted hybridoma supernatant) followed by a rabbit anti-mouse IgG-FITC secondary antibody (Dako, Hamburg, Germany, 1 : 15) or by using a mouse anti-frizzled-4 PE-conjugated antibody (Biolegend, San Diego, USA, 1 : 50). All stainings were compared according to isotype controls (rabbit IgG-APC (Miltenyi, Bergisch Gladbach, Germany, 1 : 50), mouse IgG (Biozol, Eching, Germany, 1 : 1000), and mouse IgG-PE (Biolegend, San Diego, USA, 1 : 50)), and no differences in the amount and intensity of frizzled-4 staining were observed between the two different staining procedures. Following incubation with the antibodies, cells were washed with DPBS/0.5% (v/v) BSA and centrifuged for 7 min and 210 g at 4°C. The resulting pellet was resuspended in DPBS/0.5%BSA and acquired on a FACSCanto II system (BD Bioscience, Heidelberg, Germany). Data was analysed using FlowJo software.

## 3. Results

**Figure 1:**
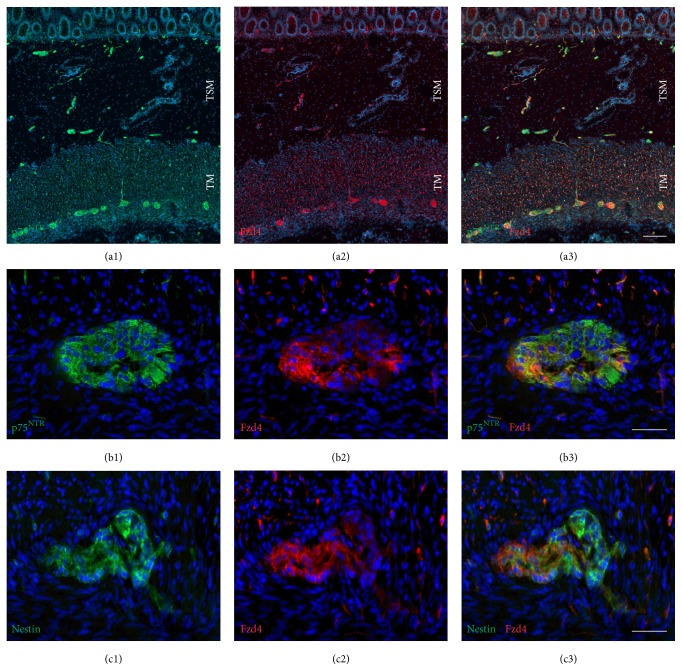
The Wnt receptor frizzled-4 is expressed in neural cells in all layers of the gut wall. Immunohistochemical analysis for frizzled-4 (Fzd4), p75^NTR^, and nestin expression on cryostat sections of human colon (sample number 1). ((a1)–(a3)) Overview of combined p75^NTR^ (green) and Fzd4 (red) immunostaining; p75^NTR^ (a1); Fzd4 (a2); merge (a3); DAPI (blue); tunica muscularis (TM); tela submucosa (TSM); scale bar: 200 *μ*m. ((b1)–(b3)) Higher magnification of combined p75^NTR^ (green) and Fzd4 (red) immunostaining of myenteric ganglia; p75^NTR^ (b1); Fzd4 (b2); merge (b3); DAPI (blue); scale bar: 50 *μ*m. ((c1)–(c3)) Combined nestin (green) and Fzd4 (red) immunostaining of myenteric ganglia; nestin (c1); Fzd4 (c2); merge (c3); DAPI (blue); scale bar: 50 *μ*m.

**Figure 2:**
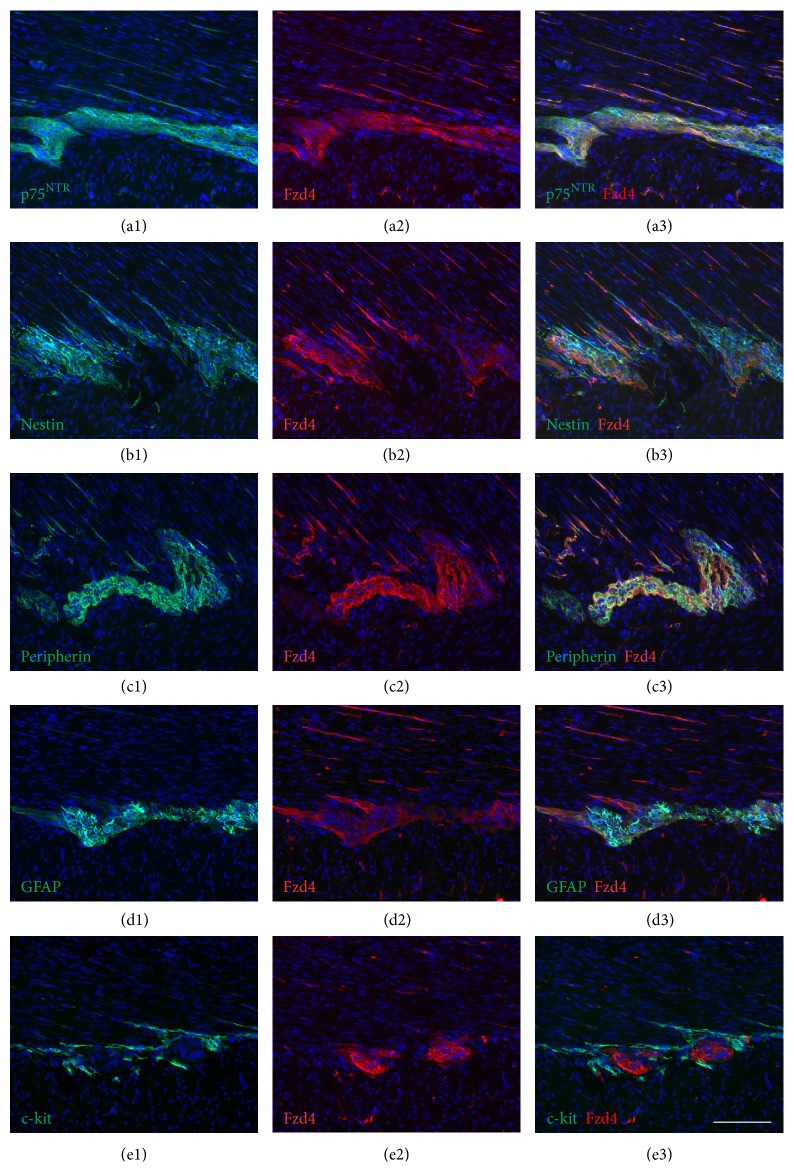
Frizzled-4 is detected in a subpopulation of neural cells. Immunofluorescence for frizzled-4 (Fzd4), p75^NTR^, nestin, peripherin, GFAP, and c-kit on cryostat sections of human ileum (sample number 2). ((a1)–(a3)) Combined p75^NTR^ (green) and Fzd4 (red) immunostaining of myenteric ganglia; p75^NTR^ (a1); Fzd4 (a2); merge (a3); DAPI (blue). ((b1)–(b3)) Combined nestin (green) and Fzd4 (red) immunostaining of myenteric ganglia; nestin (b1); Fzd4 (b2); merge (b3); DAPI (blue). ((c1)–(c3)) Combined peripherin (green) and Fzd4 (red) immunostaining of myenteric ganglia; peripherin (c1); Fzd4 (c2); merge (c3); DAPI (blue). (d1–d3) Combined GFAP (green) and Fzd4 (red) immunostaining of myenteric ganglia; GFAP (d1); Fzd4 (d2); merge (d3); DAPI (blue). ((e1)–(e3)) Combined c-kit (green) and Fzd4 (red) immunostaining of myenteric ganglia; c-kit (e1); Fzd4 (e2); merge (e3); DAPI (blue); scale bar (a–e): 100 *μ*m.

**Figure 3:**
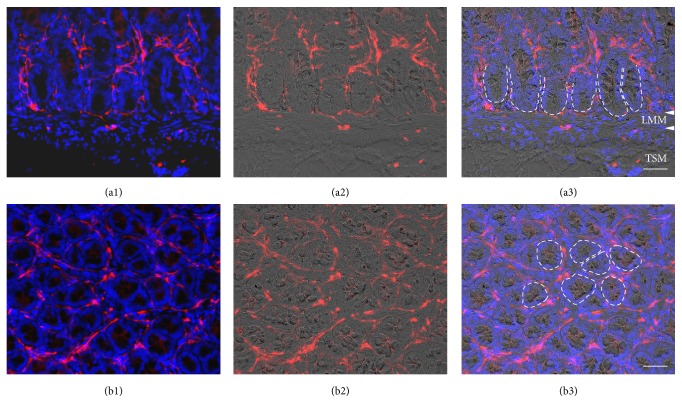
Epithelial crypts of the mucosa are surrounded by frizzled-4 positive neural extensions. Immunofluorescence of frizzled-4 (Fzd4) on cryostat sections of human ileum; (sample number 2). ((a1)–(a3)) Sagittal section. Combined Fzd4 (red) and DAPI (blue) fluorescence staining (a1); combined Fzd4 fluorescence (red) and brightfield view (a2); merge (a3). The broken lines mark the border between epithelial cells of the crypt base and the lamina propria mucosae. The white arrowheads indicate the borders of the lamina muscularis mucosae (LMM) to the lamina propria mucosae and the tela submucosa (TSM); scale bar: 50 *μ*m. ((b1)–(b3)) Horizontal section of the crypt region. Combined Fzd4 (red) and DAPI (blue) fluorescence staining (b1); combined Fzd4 (red) fluorescence and brightfield view (b2); merge (b3). The broken lines mark the border between epithelial cells of some crypts and the lamina propria mucosae; scale bar (a, b): 50 *μ*m.

**Figure 4:**
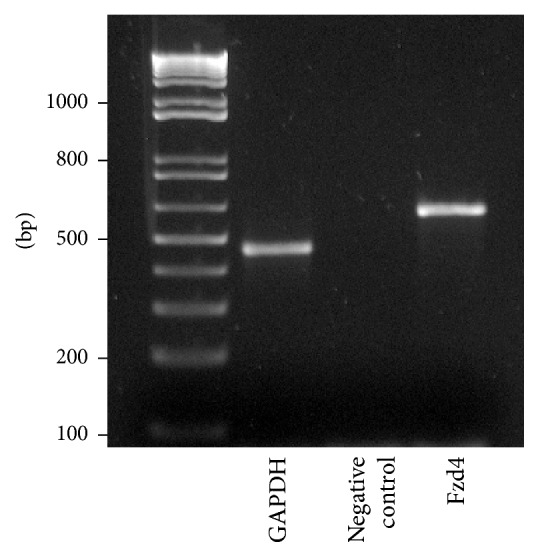
Identification of frizzled-4 (Fzd4) mRNA by RT-PCR. Total RNA was isolated from tunica muscularis. Respective primer pairs were used for detection of Fzd4 (605 bp) and the housekeeping gene GAPDH (452 bp). Isolated RNA without subsequent reverse transcriptase step served as negative control.

In the present study, we analysed the expression of the Wnt receptor frizzled-4 in the enteric nervous system of human infants aged between 4 weeks and 10 months. First, immunohistochemical staining processes for frizzled-4 glycoprotein were performed on cryostat sections from small and large intestine. Positive cells could be observed in all layers of the gut wall (Figures 1, 2, and 3). RT-PCR analysis of mRNA isolated from the tunica muscularis confirmed the presence of frizzled-4 receptor mRNA (Figure 4). To further characterize the positive cell population, coimmunostaining processes of frizzled-4 with peripherin and GFAP were performed. As shown in Figures 2 and 7 this Wnt receptor is expressed at various intensities in subpopulations of enteric neurons and glial cells (Figures 2 and 7). Frizzled-4 immunopositive neural extensions were observed in the tunica muscularis, tela submucosa, and the lamina propria mucosae (Figures 1, 2, and 3). Notably, the base of the epithelial crypts was also surrounded by frizzled-4 positive neural extensions (Figure 3). Moreover, the Wnt receptor was costained in cell populations expressing the intermediate filament nestin and the neurotrophin receptor p75^NTR^ (Figures 1, 2, and 7). Interestingly, the amount of p75^NTR^ positive cells that coexpressed frizzled-4 per ganglia was highly variable, even in the same gut area. Frizzled-4 was not found in smooth muscle cells of tunica muscularis, tunica mucosa, or the wall of blood vessels and was also absent in interstitial cells of Cajal, which were stained with c-kit (Figure 2).

In summary, we have shown by immunohistochemistry that frizzled-4 is expressed in a subpopulation of enteric nervous system cells, which are positive for peripherin, GFAP, and p75^NTR^.

**Figure 5:**
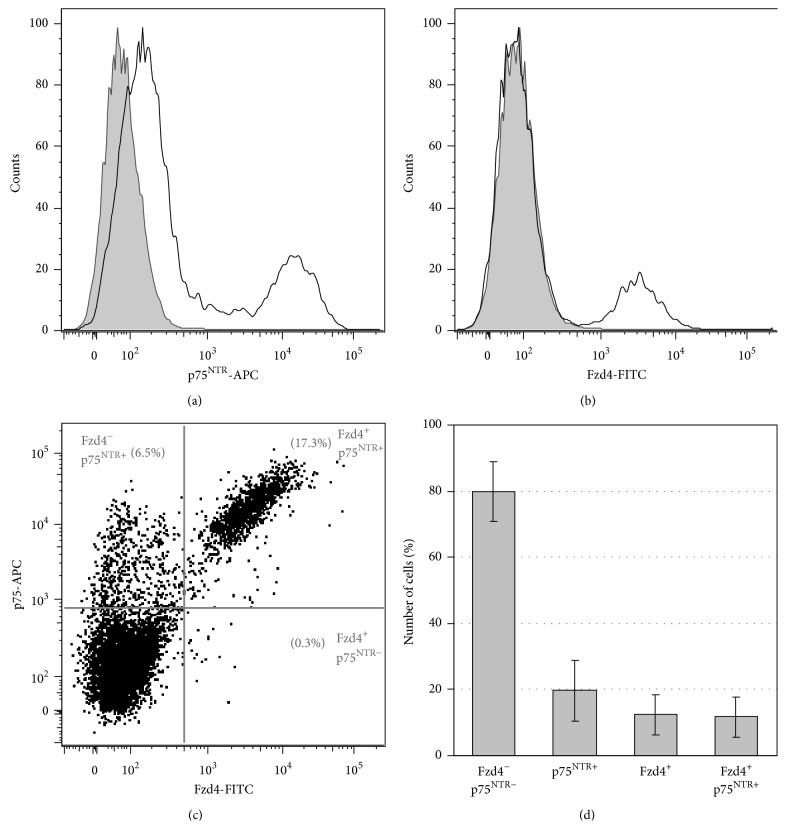
A distinct frizzled-4 positive cell population is observed by flow cytometry. Flow cytometry analysis of p75^NTR^ and frizzled-4 (Fzd4) expression on cells isolated from tunica muscularis (sample numbers 3, 5–9). (a, b) Histograms for p75^NTR^-APC and Fzd4-FITC in comparison to respective isotype controls. The isotype control staining is shown as gray filled histogram. (c) Representative dot plot of p75^NTR^-APC and Fzd4-FITC costained cells. Numbers indicate the percentage of stained cells in each gate. (d) Quantification of the percentages of unstained, p75^NTR^ Fzd4 positive, and p75^NTR^/Fzd4 costained cells from six independent experiments. Data are expressed as mean percentage ± SD of the total cell number.

In order to quantify the frizzled-4 positive cell population, enzymatically digested and immunolabeled cells isolated from tunica muscularis were stained with frizzled-4 antibody and analysed by flow cytometry (Figure 5). Using this approach, a distinct cell population could be detected in six independent experiments: 12.3%  ± 5.9% (mean ± SD) of the analysed cells stained positive for frizzled-4 in comparison to 19.7%  ± 9.2% (mean ± SD) of cells that were positive for the neurotrophin receptor p75^NTR^. Indeed, colabelling experiments demonstrated that 60.0%  ± 16.2% (mean ± SD) of p75^NTR^ positive cells expressed the Wnt receptor, whereas almost all cells found positive for frizzled-4 also expressed p75^NTR^ (93.3%  ± 8.9% (mean ± SD)). Hence, we could identify a double-positive subpopulation of p75^NTR+^ frizzled-4^+^ cells, which is clearly distinguishable from the frizzled-4 negative population.

**Figure 6:**
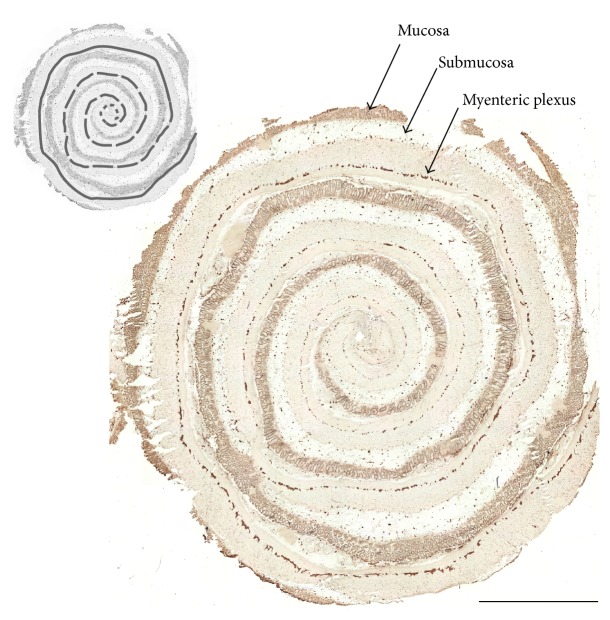
Overview of immunostained section sliced from “Swiss-rolled” tissue of Hirschsprung's disease. Cryostat section from large intestine diagnosed with Hirschsprung's disease (sample number 10) was immunostained for peripherin; scale bar: 5 mm. The center of the “Swiss-rolled” tissue marks the distal end of resected intestinal segment. In the microscopic view at the upper left corner normoganglionic, hypoglanglionic, and aganglionic areas are marked by an unbroken, broken, and dotted line, respectively.

**Figure 7:**
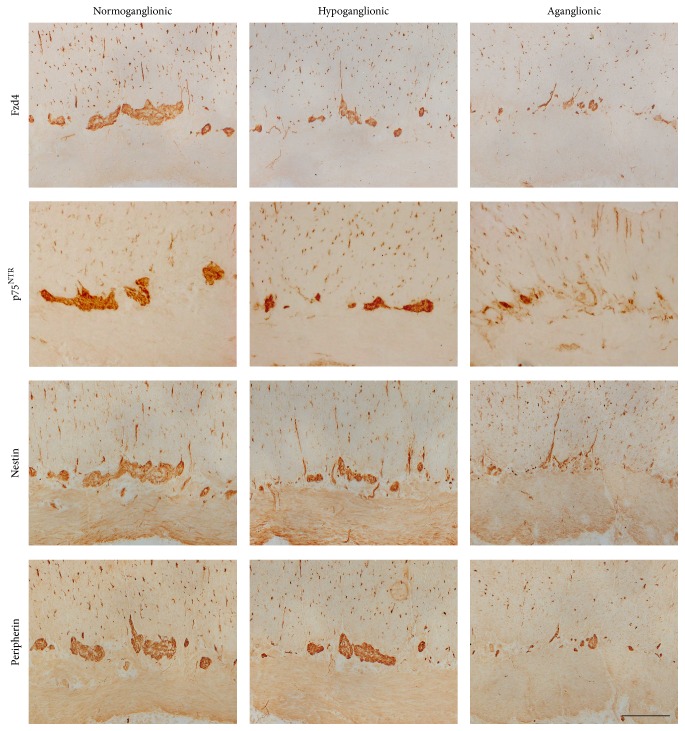
The expression of frizzled-4 in Hirschsprung's disease decreases from being oral to anal. Immunohistochemical analysis for frizzled-4 (Fzd4) in comparison to p75^NTR^, nestin, and peripherin on cryostat sections from large intestine diagnosed with Hirschsprung's disease (sample number 10). Microscopic brightfield views were taken from normoganglionic, hypoganglionic, and aganglionic areas, respectively; scale bar: 200 *μ*m.

**Figure 8:**
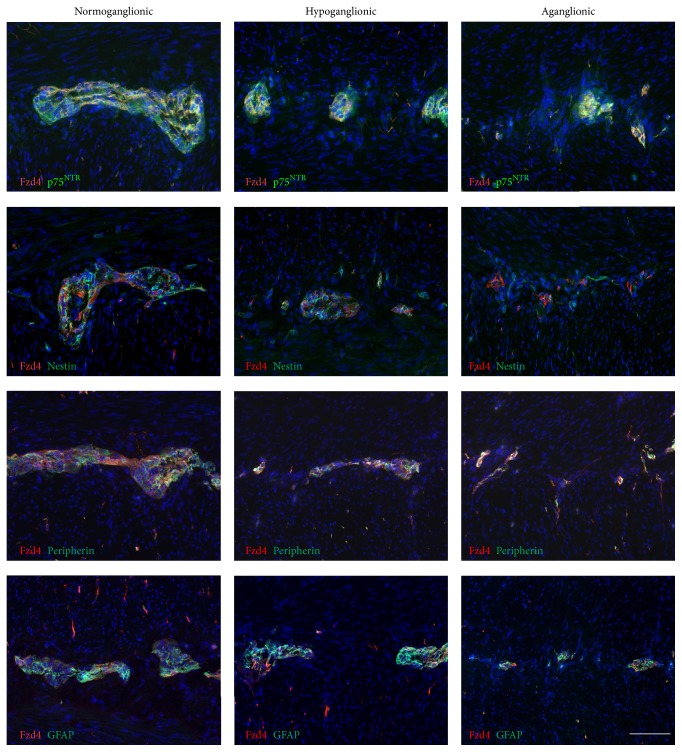
Coimmunostaining processes reveal the parallel decrease of frizzled-4 with other neural markers in Hirschsprung's disease. Immunofluorescence for frizzled-4 (Fzd4, red) in combination with p75^NTR^, nestin, peripherin, and GFAP (green) on cryostat sections from large intestine diagnosed with Hirschsprung's disease (sample number 10); cell nuclei were visualized with DAPI (blue). Fluorescence views were taken from normoganglionic, hypoglanglionic, and aganglionic area, respectively; scale bar: 100 *μ*m.

Next, we investigated the Wnt receptor expression pattern in gut samples of three patients presenting with Hirschsprung's disease. In Figure 6, a representative overview of a peripherin stained cryosection is shown, which was sliced from a “Swiss rolled” tissue of Hirschsprung's disease (Figure 6). In these sections, the size and number of ganglia typically decreased from oral to anal. The rudimentary expression of peripherin in the myenteric plexus at the anal (aganglionic) end represents peripherin expression in myenteric nerve fibers [22]. Immunohistochemical analysis of frizzled-4 in comparison to nestin, p75^NTR^, peripherin, and GFAP revealed that, in line with the other markers, the expression of frizzled-4 declined from normoganglionic to aganglionic areas of the large intestine (Figures 7 and 8).

## 4. Discussion

Wnt signalling plays an important role in the development of the peripheral nervous system. It induces and specifies the neural crest and is involved in processes regulating neural crest cell migration and formation of neuronal interconnections in the developing gut [12–14].

Among the ten identified frizzled-Wnt receptors, only frizzled-4 is able to strongly bind Norrin. Norrin is known to regulate vascular development of the inner ear and retina [23] but has also been attributed to mediate neuroprotective effects to retinal ganglion cells [20]. In humans it is associated with Norrie disease, a genetic disorder that primarily affects the eye and leads to blindness and to progressive hearing loss in a proportion of patients. Although no gastrointestinal symptoms were described in affected humans, the Norrin-frizzled-4 interaction is suspected to play an important role in colonic mucosa regeneration and colonic tumorigenesis [24].

In addition, frizzled-4 knockout mice suffer from esophageal dysfunction, apart from progressive cerebellar and auditory degeneration [25]. While the authors ascribed changes in esophageal motility rather to muscular maldevelopment and described the lower gastrointestinal ENS to be unaffected in frizzled-4 knockout mice, subtle changes in ENS architecture and the impact on the function of the gut beyond the stomach were not investigated in detail. The fact that the knockout of frizzled genes can lead to profound neurogenic abnormalities of gastrointestinal tract motility, while ENS architecture appears to be almost normal, has been impressively demonstrated by Sasselli et al. [14]. In these experiments, the loss of frizzled-3 led to developmental deficits in the ENS network and resulted in a gastrointestinal dysmotility. While the importance of Wnt signalling in neural crest and ENS development is well established, the role of this pathway in the postnatal ENS is largely unknown. Recently, preliminary data indicate an anti-inflammatory activity of Wnt signalling in the postnatal enteric nervous system of rats [26].

In our study, we analyzed the expression pattern of frizzled-4 in normal infant human gut tissue, as well as tissue collected from patients suffering from Hirschsprung's disease. Immunohistochemical analysis revealed that frizzled-4 is expressed in neural cells within the gastrointestinal tract. Subpopulations of peripherin expressing neurons and GFAP positive glial cells were stained with various intensities. In contrast, frizzled-4 was expressed neither in enteric smooth muscle nor in interstitial cells of Cajal. Interestingly, frizzled-4 expression was also observed in cell populations that stained positive for the intermediate filament nestin and the cell surface antigen p75^NTR^. Together with CD49b, these proteins have been proposed to be expressed in ENS progenitor or stem cells [18]. Indeed, some evidence supports that neurogenesis also takes place in the postnatal gut. Regeneration of the ENS has been demonstrated to take place in mice* in vivo* upon 5-HT stimulation or following injury of the gut [15–17]. In addition, the ability to isolate and propagate cells of the postnatal ENS of rodents and men indicates the persistence of progenitor or stem cell like neural cell types in the postnatal intestine [27–30]. In this context, the identification of suitable markers for isolation of these neural progenitor cells is of crucial importance for the development of cell based therapies. Intriguingly, our flow cytometry experiments showed that >93% of frizzled-4 expressing cells were also positive for p75^NTR^, whereas frizzled-4 positive cells represent only about 60% of the p75^NTR^ cell pool. Thus, the frizzled-4 antibody recognizes a subpopulation of p75^NTR^ positive cells and might represent an interesting marker for the isolation and characterization of these distinct cell populations. However, further experiments will be necessary to investigate and compare the cell biological properties of Fzd4^+^/p75^NTR+^ and Fzd4^−^/p75^NTR+^ cells.

Interestingly, our immunohistological findings demonstrate that the epithelial crypts are surrounded by frizzled-4 positive neural extension. The intestinal crypt harbors the epithelial stem compartment that is strongly regulated by the Wnt signalling pathway [31]. The close localization of ENS cell extensions to this compartment indicates that the epithelial stem cell niche might influence the enteric nervous system by secreted Wnt pathway agonists such as Wnt3a or R-spondin.

Diagnostic procedures use specific antibodies to identify diseases such as Hirschsprung's disease. However, the identification of enteric ganglion cells can be challenging in human tissue, especially if immature ganglion cells are present in newborns, where these cells can be easily confused with endothelial cells and cells of mesenchymal or immunological origin [32]. In addition, in primary diagnostic suction biopsies specimens usually only include the submucosal plexus and are often of small size, making a precise diagnosis difficult. Therefore, immunohistochemical analysis, using several antibodies targeted at various neuronal and glial antigens, has been proposed to increase sensitivity and specificity of the diagnostic procedure. In this study, we evaluated the frizzled-4 expression pattern in comparison to other cell markers in the various segments of the colon of patients suffering from Hirschsprung's disease. Immunohistochemistry was performed for p75^NTR^, nestin, GFAP, peripherin, and frizzled-4 in the normally ganglionated gut, the transition zone, and the aganglionic segment of the colon. In the examined tissues, the number of frizzled-4 positive cells declined from normoganglionic to aganglionic areas of large intestine, in accordance with the other neural markers. Whether frizzled-4 might be useful as a putative diagnostic marker for the diagnosis of Hirschsprung's disease has to be investigated in a larger cohort of patients.

## 5. Conclusion

In the present study, we analysed the expression of the Wnt receptor frizzled-4 in the enteric nervous system of human small and large intestine. Frizzled-4 was identified in a distinct subpopulation of enteric neurons and glia as well as nestin and p75^NTR^ positive cells in all layers of gut wall. In pathological human samples of Hirschsprung's disease the expression of this Wnt receptor decreased from normoganglionic to aganglionic areas of large intestine. The expression pattern of frizzled-4 indicates that the Wnt signalling pathway might be involved in the postnatal development and/or function of the enteric nervous system. Additional studies are necessary to characterize the frizzled-4 positive cell population in more detail and to elucidate the biological role of this Wnt receptor in the postnatal human enteric system.

## Figures and Tables

**Table 1:** Human gut samples.

Sample number	Gender	Patient age	Tissue	Pathology
1	Male	5 months	Colon	Normoganglionic
2	Female	2 months	Ileum	Normoganglionic
3	Female	5 months	Sigma	Normoganglionic
4	Male	2 months	Ileum	Normoganglionic
5	Male	10 months	Colon	Normoganglionic
6	Female	3 months	Sigma	Normoganglionic
7	Male	6 months	Sigma	Normoganglionic
8	Female	4 months	Ileocaecal	Normoganglionic
9	Male	7 months	Colon	Normoganglionic
10	Male	4 weeks	Colon	Hirschsprung
11	Male	6 weeks	Colon	Hirschsprung
12	Male	7 months	Colon	Hirschsprung

**Table 2:** Primary antibodies used for cell analysis.

Primary antibodies	Species	Dilution	Manufacturer
*α*-smooth muscle actin	Rabbit	1 : 100	Spring Bioscience, Pleasanton, USA
c-kit	Rabbit	1 : 500	Dako, Hamburg, Germany
Fzd4 (clone CH3A4)^*∗*^	Mouse	Undiluted	In-house (HJB)
Fzd4 PE (clone CH3A4)^*∗*^	Mouse	1 : 50	Biolegend, San Diego, USA
GFAP	Rabbit	1 : 400	Dako, Hamburg, Germany
Nestin	Rabbit	1 : 1000	Abcam, Cambridge, UK
p75^NTR^	Rabbit	1 : 500	Promega, Madison, USA
p75^NTR^ APC	Rabbit	1 : 50	Miltenyi, Bergisch Gladbach, Germany
Peripherin	Rabbit	1 : 200	Merck Millipore, Darmstadt, Germany

^*∗*^Monoclonal mouse anti-human antibody CH3A4 against frizzled-4 was raised by immunization with the retinoblastoma cell line WERI-RB-1 and specificity for frizzled-4 was verified by the selective recognition of HEK-293 cells transfected with human frizzled-4. This molecule was clustered to CD344 at the HCDM workshop in Quebec, Canada (http://www.hcdm.org/).
